# Supplementary material for: Content-rich biological network constructed by mining PubMed abstracts
Source: BMC Bioinformatics. 2004 Oct 8;5:147. doi: 10.1186/1471-2105-5-147 (PMC528731; doi:10.1186/1471-2105-5-147)
Supplement: Additional File 2 — The original results of the above study (non-essential files are deleted to keep the file size under the limit set by BMC bioinformatics). [file 1471-2105-5-147-S2.bz2 › chilibotAdditionalFile2/dip10/188ID7644522E667/html/USP_HR38.html]

 


 **USP** and **HR38** 
  
Found 5 abstracts in PubMed, retrieved 5.  
 

 What does Google say? 
 PDF only 
| .edu only 

---

**Interactive relationship** (e.g. stimulation, inhibition, etc)

**Neutral relationship**- DHR38  [ **HR38** ]  can compete in vitro against EcR for dimerization with  **USP**  and consequently disrupt EcR  **USP**  binding to an EcRE.  Ref: 7644522 Proc Natl Acad Sci U S A, 1995
- DHR38  [ **HR38** ]  also heterodimerizes with  **USP** , and this complex responds to a distinct class of ecdysteroids in a manner that is independent of EcR.  Ref: 12809604 Cell, 2003
- Although members of the NGFI B family are thought to function exclusively as monomers, we show that DHR38  [ **HR38** ]  and BHR38 in fact interact strongly with  **USP** .  Ref: 7644522 Proc Natl Acad Sci U S A, 1995
- Here, we show that a retinoid X receptor homolog, Ultraspiracle  [ **USP** ]  AaUSP, which is an obligatory partner in the functional ecdysteroid receptor, exists at the state of arrest as a heterodimer with the orphan nuclear receptor AHR38, a homolog of Drosophila DHR38  [ **HR38** ]  and nerve growth factor induced protein B.  Ref: 10637229 EMBO J, 2000

**Non-interactive relationship** (e.g. studied together, co-existance, homology, etc.)

- In addition to binding to specific response elements as a monomer, DHR38  [ **HR38** ]  interacts with the  **USP**  component of the ecdysone receptor complex in vitro, in yeast and in a cell line, suggesting that DHR38  [ **HR38** ]  might modulate ecdysone triggered signals in the fly.  Ref: 9649534 Genetics, 1998
- This response is unusual in that it does not involve direct binding of ecdysteroids to either DHR38  [ **HR38** ]  or  **USP** .  Ref: 12809604 Cell, 2003
- Interestingly, DHR38  [ **HR38** ]  is bound alone or in combination with  **USP** , providing the first evidence that the EcR  **USP**  and DHR38  [ **HR38** ]   **USP**  may directly compete for binding to a common response element.  Ref: 9466931 J Mol Biol, 1998
